# Apolipoprotein C3 genetic polymorphisms are associated with lipids and coronary artery disease in a Chinese population

**DOI:** 10.1186/1476-511X-13-170

**Published:** 2014-11-08

**Authors:** FengHe Cui, KeZhong Li, YunFeng Li, XueWu Zhang, ChangShan An

**Affiliations:** Clinical College of Yanbian University, Yanji, 133000 China; Department of Anesthesiology, Yantai Yuhuangding Hospital, Yantai, 264000 China; Department of Anesthesiology, Weihai Municipal Hospital, Weihai, 264200 China; Department of Biochemistry and Molecular Biology, Basic College of Yanbian University, Yanji, 133000 China; Department of Respiratory Medicine, Clinical College of Yanbian University, No. 1327 Juzi Street, Yanji, 133000 China

**Keywords:** Apolipoprotein C3, Gene polymorphism, Lipid, Coronary artery disease

## Abstract

**Background:**

The disorder of triglyceride (TG) metabolism leading to hypertriglyceridemia is an independent risk factor for coronary artery disease (CAD). Variants in the apolipoprotein C3 (APOC3) gene were found to be associated with elevated TG levels. The purpose of this study was to investigate the effect of two polymorphisms (1100 C/T and 3238 C/G) of *APOC3* on plasma lipid and risk of CAD in a Chinese population.

**Methods:**

The study population consisted of 600 patients with CAD and 600 age- and gender-matched controls. The *APOC3* gene polymorphism was analyzed using polymerase chain reaction-restriction fragment length polymorphism (PCR-RFLP).

**Results:**

Patients with CAD had a significantly higher frequency of *APOC3* 3238 GG genotype [odds ratio (OR) =1.64, 95% confidence interval (CI) =1.10, 2.43; *P* = 0.01] and *APOC3* 3238 G allele (OR =1.27, 95% CI =1.04, 1.55; *P* = 0.02) than controls. The findings are still emphatic by the Bonferroni correction. When stratifying by hyperlipidemia, CAD patients with hyperlipidemia had a significantly higher frequency of *APOC3* 3238 GG genotype (OR =1.73, 95% CI =1.13, 2.64; *P* = 0.01) than without hyperlipidemia. The *APOC3* 3238 G allele was significantly associated with increasing plasma TG levels and very-low-density lipoprotein cholesterol (VLDL-C) levels both in cases and controls (*P* < 0.001).

**Conclusions:**

The *APOC3* 3238 G allele might contribute to an increased risk of CAD as a result of its effect on TG and VLDL-C metabolism.

## Introduction

Coronary artery disease (CAD), one of the most common cardiovascular diseases, is the leading cause of morbidity and mortality worldwide [1]. It is caused by multiple interacting endogenous and exogenous factors [2]. There are several emerging risk factors for CAD including smoking, obesity, stress, diabetes, lack of exercise, high alcohol consumption, and hyperlipidemia [3]. Genetic risk, estimated to account for 40-60% of susceptibility to CAD, has until recently been unknown [4–7]. Recently, genome-wide association studies (GWAS) identified a series of single-nucleotide polymorphisms (SNPs) associated with CAD [8–10].

Apolipoprotein C3 (APOC3), a component of triglyceride (TG)-rich lipoproteins and high-density lipoprotein (HDL), was mainly synthesized in the liver and to some extent in the intestine [11]. *APOC3* gene, mapped to chromosome 11q23, was involved in transport and clearance of chylomicron remnants, and very-low-density lipoprotein (VLDL) and HDL from the bloodstream [12, 13]. Two common SNPs have been identified in the *APOC3* gene: 1100 C/T and 3238 C/G [14, 15]. The *APOC3* 3238 C/G polymorphisms have been found to be associated with altered plasma TG concentrations [16].

Recently, case–control study has suggested that the *APOC3* 3238G allele was a risk factor for CAD in Indians [17–19]. However, no other studies have confirmed this finding, and no similar studies were represented in Chinese. The purpose of this study was to investigate the effect of two polymorphisms (1100 C/T and 3238 C/G) of *APOC3* on plasma lipid and risk of CAD in a Chinese population.

## Materials and methods

### Study population

A hospital-based case–control study was carried out in 600 patients with CAD and 600 age- and gender-matched controls from January 2012 to January 2014 in the Yantai Yuhuangding Hospital and Clinical College of Yanbian University, China. Based on WHO criteria, CAD cases were defined as those having severe angiostenosis (>50%) in at least one major coronary artery determined by angiography. The control group was composed of age- and gender-matched subjects who had undergone a coronary angiography in the same recruitment period as the CAD patients, without angiographic evidence of CAD. Controls selected also had at least one conventional predisposing factor of CAD. The controls were required to have no history or ECG signs of angina pectoris, myocardial infarction, or other cardiovascular diseases. In addition, similar to the cases the controls were all required to be born in China to native Chinese Han parents. To confirm the diagnosis, two physicians reviewed the hospital records and validated each case. The Institutional Ethical Committee approved this study, and all participants gave written informed consent according to the Declaration of Helsinki.

### DNA extraction and genotyping

The commercially available Qiagen kit (QIAGEN Inc., Valencia, CA, USA) was used to extract DNA from peripheral blood leukocytes. Genomic DNA was isolated from 20 g/L ethylenediaminetetraacetic acid (EDTA) or sodium citrate anticoagulated 3–5 ml venous blood and stored at 4°C. Polymerase chain reaction restriction fragment length polymorphism (PCR-RFLP) assay was applied to assess the *APOC3* gene polymorphisms. Based on the GenBank reference sequence, the PCR primers designed for *APOC3* 1100 C/T and 3238 C/G were 5′-AGA GGC CGA TCC ACC CCA CTC AGC C-3′ (forward) and 5′-GGC GGT CTT GGT GGC GTG CTT CAG G-3′ (reverse); 5′-CAT GGT TGC CTA CAG AGG AGT-3′ (forward) and 5′-TGA CCT TCC GCA CAA AGC TGT-3′ (reverse), respectively. The amplified PCR products were digested with *Sst*I (3238 C/G) and *Sac*I (1100 C/T) (New England BioLabs, Missisauga, ON, Canada). Details of PCR conditions have been described elsewhere [20]. The digested PCR products were resolved on a 3% agarose gel and stained with ethidium bromide for visualization under UV light. For quality control, two independent observers, read all genotypes without knowing about the case or control status. Direct DNA sequencing was performed to confirm the genotyping results.

### Statistical analysis

All statistical tests were performed using the program SPSS (SPSS Inc., Chicago, IL). Linkage disequilibrium was calculated by EXCEL and the method of Chakravarti et al. [21]. Hardy–Weinberg equilibrium was assessed by the *x*^2^ goodness-of-fit test. Differences between continuous variables were assessed by Student’s *t* test, while those between categorical variables were evaluated using Pearson *x*^2^ test. The existence of differences in genotypic frequencies between groups was assessed by means of Pearson *x*^2^ test and calculating the odds ratio (OR) with the 95% confidence intervals (CI). OR adjusted for other covariables (hypertension, diabetes, smoking, age and gender) were determined using logistic regression models. We also use the Bonferroni correction for total number of independent comparisons. A *P*-value was considered significant at a level of <0.05.

## Results

### Characteristics of participants

General characteristics of cases and controls were showed in Table 1. No significant differences were found between the CAD cases and controls in age, gender, family history, hypertension, ApoA1 and ApoB (Table 1). Univariate study was performed to identify that smoking status (*P* < 0.001), diabetes (*P* < 0.001), obesity (*P* < 0.001), hyperlipidemia (*P* < 0.001), TG (*P* < 0.001), TC (*P* < 0.001), HDL-C (*P* = 0.002), LDL-C (*P* = 0.001) and VLDL-C (*P* < 0.001) were risk factors of CAD (Table 1). Hardy–Weinberg proportions were observed for all the SNPs. The 1100 C/T and 3238 C/G in the APOC3 gene are in complete linkage disequilibrium in the Chinese population (Table 2).

**Table 1:** **General characteristics of cases and controls**

	CAD	Controls	***P***
Number of subjects	600	600	
Age (years), mean(SD)	57.3 ± 10.8	56.9 ± 10.1	0.51
Gender (Male/Female)	486/114	462/138	0.09
Smoking status (Ever/Never)	192/408	98/502	<0.001
Family history (Positive/Negative)	98/502	77/523	0.09
Hypertension (Positive/Negative)	305/295	280/320	0.15
Diabetes (Positive/Negative)	156/444	78/522	<0.001
Obesity (Positive/Negative)	176/424	85/515	<0.001
Hyperlipidemia (Positive/Negative)	188/412	92/508	<0.001
TG (mg/dL)	180.5 ± 92.3	151.7 ± 79.4	<0.001
TC (mg/dL)	202.1 ± 60.2	189.6 ± 47.9	<0.001
HDL-C (mg/dL)	37.4 ± 11.3	39.5 ± 12.4	0.002
LDL-C (mg/dL)	124.8 ± 49.8	116.7 ± 35.4	0.001
VLDL-C (mg/dL)	39.8 ± 23.7	32.7 ± 19.5	<0.001
ApoA1 (mg/dL)	127.3 ± 23.7	126.8 ± 22.9	0.71
ApoB (mg/dL)	90.6 ± 28.7	89.7 ± 24.5	0.56

*Abbreviations:* *CAD* coronary artery disease, *TG* triglyceride, *TC* total cholesterol, *HDL-C* high-density lipoprotein cholesterol, *LDL-C* low-density lipoprotein cholesterol *VLDL-C* very-low-density lipoprotein cholesterol, *Apo* apolipoprotein.

**Table 2:** **Genotype and allele frequencies of APOC3 gene polymorphisms among coronary artery disease cases and healthy controls**

Genotypes	Cases (%)	Controls (%)	OR (95% CI)	***P***
1100 CC	224(37.3)	235(39.2)	1.00(Reference)	
1100 CT	202(33.7)	215(35.8)	0.99(0.76,1.29)	0.92
1100 TT	174(29.0)	150(25.0)	1.22(0.92,1.62)	0.18
1100 C allele frequency	650(54.2)	685(57.1)	1.00(Reference)	
1100 T allele frequency	550(45.8)	515(42.9)	1.13(0.96,1.32)	0.15
3238 CC	414(69.0)	433(72.2)	1.00(Reference)	
3238 CG	114(19.0)	121(20.2)	0.99(0.74,1.32)	0.92
3238 GG	72(12.0)	46(7.6)	1.64(1.10,2.43)	0.01
3238 C allele frequency	942(78.5)	987(82.3)	1.00(Reference)	
3238 G allele frequency	258(21.5)	213(17.7)	1.27(1.04,1.55)	0.02

### APOC3 3238 C/G polymorphisms, lipids and CAD

Patients with CAD had a significantly higher frequency of *APOC3* 3238 GG genotype (OR =1.64, 95% CI =1.10, 2.43; *P* = 0.01) and *APOC3* 3238 G allele (OR =1.27, 95% CI =1.04, 1.55; *P* = 0.02) than controls (Table 2). The findings are still emphatic by the Bonferroni correction. When stratifying by hyperlipidemia, CAD patients with hyperlipidemia had a significantly higher frequency of *APOC3* 3238 GG genotype (OR =1.73, 95% CI =1.13, 2.64; *P* = 0.01) than without hyperlipidemia (Table 3). The *APOC3* 3238 G allele was significantly associated with increasing plasma TG levels and VLDL-C levels both in cases and controls (*P* < 0.001) (Table 4).

**Table 3:** **Stratification analysis of APOC3 3238 C/G polymorphisms in coronary artery disease cases**

	Cases	CC			CG			GG		
		n (%)	OR (95% CI)	***P***	n (%)	OR (95% CI)	***P***	n (%)	OR (95% CI)	***P***
Smoking status	600	414(69.0)	1(Reference)		114(19.0)	1(Reference)		72(12.0)	1(Reference)	
Ever	192	133(69.3)	1.00(0.78,1.29)	0.98	35(18.2)	0.96(0.64,1.45)	0.84	24(12.5)	1.04(0.64,1.70)	0.87
Never	408	281(68.9)	0.99(0.82,1.22)	0.99	79(19.4)	1.02(0.75,1.39)	0.91	48(11.7)	0.98(0.67,1.44)	0.92
Diabetes	600	414(69.0)	1(Reference)		114(19.0)	1(Reference)		72(12.0)	1(Reference)	
Positive	156	107(68.6)	0.99(0.75,1.31)	0.97	30(19.2)	1.01(0.65,1.57)	0.96	19(12.2)	1.02(0.59,1.73)	0.96
Negative	444	307(69.1)	1.00(0.83,1.21)	0.98	84(18.9)	1.00(0.73,1.35)	0.98	53(11.9)	0.99(0.68,1.45)	0.98
Obesity	600	414(69.0)	1(Reference)		114(19.0)	1(Reference)		72(12.0)	1(Reference)	
Positive	176	120(68.2)	0.99(0.76,1.29)	0.93	35(19.9)	1.05(0.69,1.58)	0.83	21(11.9)	0.99(0.60,1.66)	0.98
Negative	424	294(69.3)	1.01(0.83,1.22)	0.96	79(18.6)	0.98(0.72,1.34)	0.90	51(12.0)	1.00(0.69,1.47)	0.99
Hyperlipidemia	600	414(69.0)	1(Reference)		114(19.0)	1(Reference)		72(12.0)	1(Reference)	
Positive	188	117(62.2)	0.90(0.69,1.17)	0.44	32(17.0)	0.90(0.59,1.37)	0.61	39(20.7)	1.73(1.13,2.64)	0.01
Negative	412	297(72.1)	1.05(0.86,1.27)	0.66	82(19.9)	1.05(0.77,1.43)	0.77	33(8.0)	0.67(0.43,1.03)	0.07

**Table 4:** **Lipid profiles of coronary artery disease cases and controls according to APOC3 3238 C/G polymorphisms**

	CAD			***P***value	Controls			***P***value
	CC	CG	GG		CC	CG	GG	
TG (mg/dL)	162.3 ± 86.5	197.6 ± 90.4	253.9 ± 104.7	<0.001	138.7 ± 75.8	170.5 ± 80.6	224.6 ± 95.7	<0.001
TC (mg/dL)	199.4 ± 58.5	215.4 ± 62.7	196.3 ± 58.4	0.65	182.3 ± 47.1	213.8 ± 49.8	194.4 ± 48.7	0.74
HDL-C (mg/dL)	37.5 ± 11.1	40.8 ± 10.4	31.5 ± 9.8	0.56	37.4 ± 11.7	46.2 ± 14.5	41.7 ± 12.4	0.52
LDL-C (mg/dL)	127.1 ± 49.8	128.8 ± 49.8	105.7 ± 42.3	0.09	115.3 ± 34.9	130.6 ± 37.4	104.5 ± 32.7	0.34
VLDL-C (mg/dL)	34.8 ± 19.8	45.8 ± 24.1	59.1 ± 27.4	<0.001	29.6 ± 18.7	37.0 ± 20.1	48.2 ± 21.3	<0.001
ApoA1 (mg/dL)	124.6 ± 22.9	138.4 ± 25.6	125.2 ± 23.4	0.78	124.5 ± 22.1	135.6 ± 24.1	124.7 ± 21.8	0.68
ApoB (mg/dL)	88.3 ± 28.1	99.8 ± 30.1	89.1 ± 27.9	0.73	87.3 ± 24.2	97.7 ± 28.7	91.4 ± 25.7	0.82

### APOC3 1100 C/T polymorphisms, lipids and CAD

No association was found between *APOC3* 1100 C/T polymorphisms and CAD (Table 2). When stratifying by smoking status, diabetes, obesity and hyperlipidemia, no significant differences were found in any groups (Table 3).

## Discussion

A lot of studies have been conducted to examine the association of genetic polymorphism and risk of CAD. A meta-analysis of 9 studies that included 1,700 CAD patients and 4,081 healthy controls suggested that *ALDH2* Glu504Lys polymorphism may be associated with increased risk of CAD and myocardial infarction in East Asians, especially among Chinese and Korean populations [22]. A meta-analyses of 26 studies that included 12,776 cases and 6,371 controls found that -1562C>T polymorphism in the promoter region of matrix metalloproteinase-9 may have association with CAD risk in Asian populations [23]. A meta-analyses of 22 studies including 3,502 CAD patients and 3,071 controls suggested that the angiotensin II receptor, type 1 gene A1166C polymorphism might be a genetic marker for the development of CAD in Chinese populations, especially in the context of studies with northern and older subjects [24, 25]. A meta-analyses of 11 studies involving 22,584 subjects showed that *PTGS2* -765G/C was associated with a decreased risk of CAD [26]. A meta-analyses of ten studies with 4,413 patients found that apolipoprotein M T-778C polymorphism was associated with serum lipid levels and the risk of CAD in the Chinese population [27]. A meta-analyses of 11 studies involving 5,535 CAD patients and 5,626 controls indicated that the Connexin37 C1019T polymorphism may be a moderate risk factor for CAD [28]. A meta-analyses of 20 studies comprising 15,591 participants found that apolipoprotein C3 Sst I and T-455C polymorphisms might be associated with CAD risk [14]. A meta-analyses of 9 studies that included 3,439 cases and 14,182 controls suggested that Cyclooxygenase-2 (COX-2) rs20417 polymorphism may contribute to CAD development, especially in Asians [29]. A prospective case–control study found that the rsl2221497 polymorphism in liver X receptor alpha gene was associated with susceptibility of CAD in Han population [30]. A meta-analyses of 40 studies including 4,564 CAD cases and 3,985 controls suggested an association between apolipoprotein E (ApoE) epsilon4 allele and increased risk of CAD in Chinese population [31].

The *APOC3* gene polymorphisms were also associated with many other diseases. The polymorphisms -482 C/T and -455 T/C in *APOC3* were associated with nonalcoholic fatty liver disease and insulin resistance [32]. A case–control study suggested that *APOC3* 1100 C/T was associated with increased risk of diabetes probably through mechanisms other than direct effects on TG [33]. A population-based prospective cohort (n =7,983) *APOC3* -482T allele had increased type 2 diabetes risk [34]. *APOC3* gene polymorphisms contributed to an unfavorable lipid profile in patients with HIV [13]. A case–control study suggested that the minor alleles of *APOC3* -455 T/C polymorphisms were closely associated with acute coronary syndrome [35]. A case–control study suggested that *APOC3* -482TT genotype was independently associated with elevated fasting triglyceride concentrations in obese men [36]. A case–control study involving 374 Chinese type 2 diabetic patients with and 392 without diabetic nephropathy found that the hepatic lipase -514C/T polymorphism interaction with polymorphisms in *APOC3* -482C/>T increased the risk of diabetic nephropathy [37]. A case–control study suggested that *APOC3* SstI polymorphism was weakly associated with sporadic Alzheimer's disease in a Chinese population [38]. A case–control study suggested that *APOC3* promoter polymorphisms (-482 C/T and -455 T/C) were associated with the metabolic syndrome [39, 40].

Although the exact mechanism by which the *APOC3* 3238 G allele might contribute to an increased risk of CAD is still unclear, it is widely suspected that as a result of its effect on lipid metabolism. A meta-analyses of 29 Western prospective studies involving 262,525 participants indicated moderate and highly significant associations between triglyceride values and CAD risk [41]. The Bogalusa Heart Study found that *APOC3* 3238 C/G polymorphisms were associated with higher serum triglyceride levels [42]. Our study also found that the *APOC3* 3238 G allele was significantly associated with increasing plasma TG levels and VLDL-C levels.

Strength of this study was a relatively large sample size. Several limitations of our study should be addressed. First of all, potential selection bias might have been present, because this is a hospital based case control study and the subjects may not be representative of the general population. Second, this study only considers a Chinese population that may limit the application of these findings to other ethnic populations. Third, CAD is induced by multiple genes and environmental factors, which were not explored in the present study.

## Conclusion

In conclusion, our study suggested that the *APOC3* 3238 G allele might contribute to an increased risk of CAD as a result of its effect on TG and VLDL-C metabolism. Additional studies are needed to confirm this finding.
